# Application of the transtheoretical model to sedentary behaviors and its association with physical activity status

**DOI:** 10.1371/journal.pone.0176330

**Published:** 2017-04-27

**Authors:** Ho Han, Kelley Pettee Gabriel, Harold Willis Kohl

**Affiliations:** 1 Department of Kinesiology and Health Education, University of Texas at Austin, Austin, TX, United States of America; 2 Epidemiology, Human Genetics, and Environmental Sciences, University of Texas School of Public Health, Austin, TX, United States of America; Vanderbilt University, UNITED STATES

## Abstract

**Background:**

The Transtheoretical Model (TTM) is a successful framework for guiding behavior change programs for several health behaviors, yet its application to reduce of sedentary behavior has been neglected. In addition, no data exist regarding the association between determinants of sedentary behaviors based on the TTM and physical activity behavior. The purpose of this study was to investigate college students’ stages of motivational readiness to avoid sedentary behaviors and relevant psychological determinants using newly developed TTM questionnaires and to identify the association between current physical activity and sedentary behaviors based on TTM constructs.

**Methods:**

Data were obtained from 225 college students enrolled in health education and physical education courses. Participants completed a package of questionnaires including validated TTM, physical activity and sitting time questionnaires. Participants also wore an accelerometer for seven consecutive days. MANOVAs were conducted to determine mean differences in psychological constructs across the TTM stages, and Chi-square tests and Spearman correlation were used to evaluate the associations between current physical activity and sedentary behavior.

**Results:**

A majority of the participants were in the sedentary stages, and men and women differed in proportion of individuals in the stages (78.0% vs. 68.1%, respectively). The gender difference was also found in use of the processes of change. In general, the mean scores of the TTM constructs increased as the stages progressed. No significant associations were found between the TTM constructs for sedentary behavior and current physical activity levels (*p*>0.05).

**Conclusions:**

A high proportion of college students were in sedentary stages regardless of physical activity levels, but different distributions in men and women. Participants in earlier stages were less likely to utilize the TTM constructs to reduce sedentary behaviors than those in later stages. A lack of association between physical activity and the psychological determinants of sedentary behavior was found.

## Introduction

In addition to various health benefits of physical activity, consistent evidence has been presented of the possible deleterious health consequences of prolonged sitting time independent of current physical activity level [1–3]. Sedentary behaviors have been defined as any waking behaviors in a posture of sitting, reclining, or lying down expending little energy (i.e., 1.0 to 1.5 metabolic equivalents) such as watching TV, using a computer, playing a video game, reading or listening [4, 5]. In contrast, physical activity is a behavior that involves human movement, resulting in physiological attributes including increased energy expenditure and improved physical fitness [6], and an individual can achieve the minimal physical activity recommendation for substantial health benefits with engagement in 150 min a week (i.e., preferably, spread throughout the week) of moderate-intensity physical activity [7]. In this perspective, an active individual (e.g., meeting the physical activity guidelines) can be also mostly sedentary in the remaining exercise time of the day, and thus sedentary behavior is distinct from lack of physical activity [8]. According to a recent study, U.S. adolescents ages 16–19 years and young adults (ages 20–29 years) spent more than half of their waking time, or 8.0 and 7.5 hours/day, respectively, in sedentary pursuits [5].

Evidence-based approaches for addressing sedentary behaviors may help develop effective interventions to reduce such behaviors and help identify determinants of sedentary behaviors. The Transtheoretical Model (TTM) is an integrative model designed to assess an individual’s readiness to change a behavior. It is also used to provide strategies to guide the individual to change the behavior through stages of motivational readiness [9]. This model has been effectively applied to various health behaviors such as smoking, alcohol use, substance abuse, and physical activity [10–13]. Many such intervention studies using the TTM have shown greater effectiveness with stage-matched interventions in changing behavior compared to non-stage matched programs [14]. Despite the convincing evidence that the TTM has been successfully applied to a variety of health behaviors, no studies have applied the model to sedentary behaviors as an independent health risk behavior. The application of the TTM to sedentary behaviors may provide crucial and in-depth information about participants’ readiness to change their sedentary behaviors and strategies for reducing their sedentary time.

The association between physical activity and sedentary behaviors is still unclear. Some researchers support the displacement hypothesis that posits an inverse relationship between physical activity and sedentary behaviors [15, 16]. For example, the more time an individual engages in sedentary behaviors, the less time the individual will participate in physical activity or vice versa. In contrast, others, such as Whitfield *et al*.[17], propose a different idea about the relationship in which sedentary behaviors are distinct from a lack of physical activity: an individual participating in a high amount of physical activity can still spend a substantial amount of time engaged in sedentary behaviors. These controversial findings require further research providing more information about the relationship between physical activity and sedentary behaviors.

Identifying the association between current physical activity levels and the TTM constructs for sedentary behaviors may help clarify the ambiguous relationship between physical activity and sedentary behaviors in different perspectives (i.e., psychosocial determinants of sedentary behaviors and physical activity) and to identify potential intervention strategies targeting both behaviors (i.e., changes in physical activity following interventions to reduce sedentary times) [18]. Therefore, the purposes of the present study are to investigate college students’ stages of motivational readiness to avoid sedentary behaviors and relevant psychological determinants using the TTM and to identify the association between current physical activity levels and sedentary behaviors based on TTM constructs.

## Materials and method

### Participants and protocol

A convenience sample of 225 college students (116 men and 109 women), aged 18 to 24 years, was recruited from health education and physical education courses offered in the Fall 2014 at the University of Texas at Austin. The participants represented various academic disciplines on campus (e.g., engineering, education, natural sciences). All participants were provided and signed an informed consent prior to participation. Study participation was totally voluntary and anonymous. The protocol was approved by the Institutional Review Board of the University of Texas at Austin.

After informed consent, participants were given a triaxial accelerometer (ActiGraph GT3X+; Pensacola, FL) with written instructions and asked to wear the monitor on their right hip during all waking hours for seven consecutive days. On the day of device return, participants completed a package of questionnaires (S1 Questionnaire) including questions collecting demographic information, a previously developed TTM questionnaire for sedentary behaviors [19], a self-administered version of the past-week Modifiable Activity Questionnaire (SMAQ) [20], and a Multi-context Sitting Time Questionnaire (MSTQ) [21].

### Instruments

#### Stages of motivational readiness to avoid sitting time

A newly developed Stages of Motivational Readiness Questionnaire was utilized to identify participants’ motivational readiness to avoid sitting time. The questionnaire consists of a single question with a five-item, dichotomous (yes/no) or (true/false) response options and differentiates the participants’ intentions to avoid sitting time into one of the five stages: Precontemplation, Contemplation, Preparation, Action, and Maintenance. Validation of the questionnaire has indicated strong concurrent (χ2 = 25.0, *p*<0.001) and construct (*p*<0.01) validities and strong internal consistency (*k* = 0.62) and test-retest reliabilities (Cronbach’s alpha ranging from 0.72 to 0.88) [19].

#### Other TTM constructs for avoiding sitting time

A Processes of Change Questionnaire for avoiding sitting time was used to determine ten processes that were used to reduce sedentary behaviors by individuals at the different stages. The questionnaire consists of 40 items including a set of 4 items assessing each of the 10 processes of change. Participants were asked how frequently they used each of the 10 processes on a 5-point Likert scale from 1 (never) to 5 (repeatedly). Strong Internal consistency (Cronbach’s alpha ranging from 0.73 to 0.87) and intraclass correlation coefficients (ICC) (0.72 to 0.94) of this measurement were indicated [19]. Self-efficacy was measured using a 6-item situational confidence scale modified for avoiding sedentary behaviors. Participants were asked how confidently they can avoid prolonged sitting time in some situations leading to be sedentary. The items were scored on a 5-point Likert scale from 1 (not at all confident) to 5 (extremely confident). Internal consistency and ICC of this questionnaire were 0.75 and 0.72, respectively [19]. Lastly, the Decisional Balance Questionnaire consisting 6 pros and cons each for being sedentary was used to identify how important each statement of pros and cons was with respect to the individual’s decision of whether to avoid sitting time or not. The questionnaire was a 5-point Likert scale from 1 (not at all important) to 5 (extremely important). Internal consistency and ICC for this scale were 0.76 and 0.87, respectively [19].

#### Past-week modifiable activity questionnaire

The self-administered past-week Modifiable Activity Questionnaire (SMAQ) was used to assess leisure time physical activity over the past 7 days. The SMAQ included 38 common leisure physical activities (e.g., swimming, walking, football, basketball) in this population. Times of leisure time physical activity were assessed for any reported activities in hours/week, weighted by estimated metabolic equivalent (MET) of that activity [22] and summed for all performed activities. Total leisure time physical activity estimates were expressed as MET hours per week (MET·hours/week) [23]. The past-week Modifiable Activity Questionnaire, from which the SMAQ was developed, has been shown to be reliable and valid [20, 24]. For self-report physical activity estimates, the threshold for meeting or not meeting 2008 Physical Activity Guidelines was 7.5 MET·hours/week.

#### Multi-context Sitting Time Questionnaire (MSTQ)

This questionnaire, developed by Whitfield et al. [21], was used to assess time spent in sitting across domains and contexts. Although this questionnaire was originally developed to apply to both students and professionals, some contexts were modified for student-focused questions. Strong test-retest reliability (r > 0.70) and convergent validity (r = 0.34–0.61) for the MSTQ was reported by the authors [21].

#### Objective measures of physical activity and sedentary time

Objective measurement was used to assess times spent in sedentary behaviors and physical activity using a triaxial accelerometer (ActiGraph GT3X+; Pensacola, FL) (S1 Table). Participants were given an instruction sheet and asked to wear the device for seven consecutive days. A 1-second epoch was reported, and the collected data were screened for wear time by using a validated cut-point requiring a minimum of 10 hours of wear time per day for at least 4 of 7 days [25]. Device non-wear time was detected using Troiano algorithm (i.e., 60 consecutive minutes of 0 activity counts, with an allowance for 1–2 min of detected counts between 0 and 100 [26]. Wear time was calculated by subtracting derived non-wear time from 24 h [26]. Total sedentary time was calculated as the amount of time accumulated below 100 counts per minutes during a period of wearing time [5]. In addition, sedentary time was adjusted for total wear time to attenuate the difference in absolute sedentary time occurred by different wear times across study participants. Freedson cut-point [27] were used to classify accelerometer counts into minutes per day of light- (100–1951 count per minute), moderate- (1952–5724 counts per minute), and vigorous- (≥5725 counts per minute) intensity physical activity. To compare with physical activity recommendations, a modified activity bout was defined as ten or more consecutive minutes above the moderate-intensity threshold (≥1952 counts per minute) with allowance of 2 minutes below the threshold [25]. The cut-point of meeting the guidelines was determined as at least 150 minutes a week of moderate-intensity, 75 minutes a week of vigorous-intensity, or an equivalent combination of moderate- and vigorous-intensity aerobic physical activity [7].

### Data analysis

Descriptive statistics were computed for measured parameters including demographics and relevant variables presented as means, medians, standard deviations, frequencies, and percentages. All variables were assessed for normality with a Shapiro-Wilk test. Due to non-normally distributed sedentary times, medians with inter-quartile range (IQR) were reported for presenting unadjusted minutes of sedentary behaviors across the stages, and men and women differences for each stage were tested with the Mann-Whitney U tests and independent-sample *t* tests as appropriate. Multivariate analyses of variance (MANOVAs) with post-hoc pairwise comparisons were conducted to determine mean differences in processes of change, self-efficacy, and decisional balance across the stages and to compare scores of the TTM constructs between two groups of meeting and non-meeting physical activity guidelines. In order to identify the relationships between physical activity and sedentary behaviors, (1) Chi-square tests, (2) Spearman correlations, and (3) MANOVAs were conducted for categorical variables (i.e., stages of motivational readiness for sedentary behaviors and meeting/non-meeting physical activity guidelines), continuous variables (i.e., minutes of physical activity and minutes of sedentary behaviors), and both (scores of TTM outcomes for sedentary behaviors and meeting/non-meeting the guidelines), respectively. All statistical analyses were conducted using IBM SPSS 20 for Windows.

## Results

Descriptive characteristics of the participants are presented in Table 1. This sample included 116 men and 109 women with a mean age (± sd) 20.4 (± 1.8) years. Average body mass index (BMI) was 23.7 (±3.2) kg/m^2^. In general, the participants were evenly distributed among college year (i.e., 1^st^ through 4^th^ year) and ethnicity (i.e., White, Hispanic, African American, and Asian) categories except for relatively low numbers of the first year students (4.5%) and African American students (10.2%). The low proportion of African American students in this study was similar to its proportion (7.2%) of the entire undergraduate students at the university.

**Table 1:** Demographic characteristics.

Characteristic	Frequency (%) or Mean (SD)
Age	20.4 (1.8)
Gender	
Men	116 (51.6)
Women	109 (48.4)
College Years	
1	10 (4.5)
2	83 (36.9)
3	68 (30.2)
4	64 (28.4)
Ethnicity	
White	81 (36.0)
Hispanic	66 (29.3)
African American	23 (10.2)
Asian	55 (24.5)
BMI	23.7 (3.2)

### Application of the TTM for avoiding sedentary behaviors

**Fig 1:**
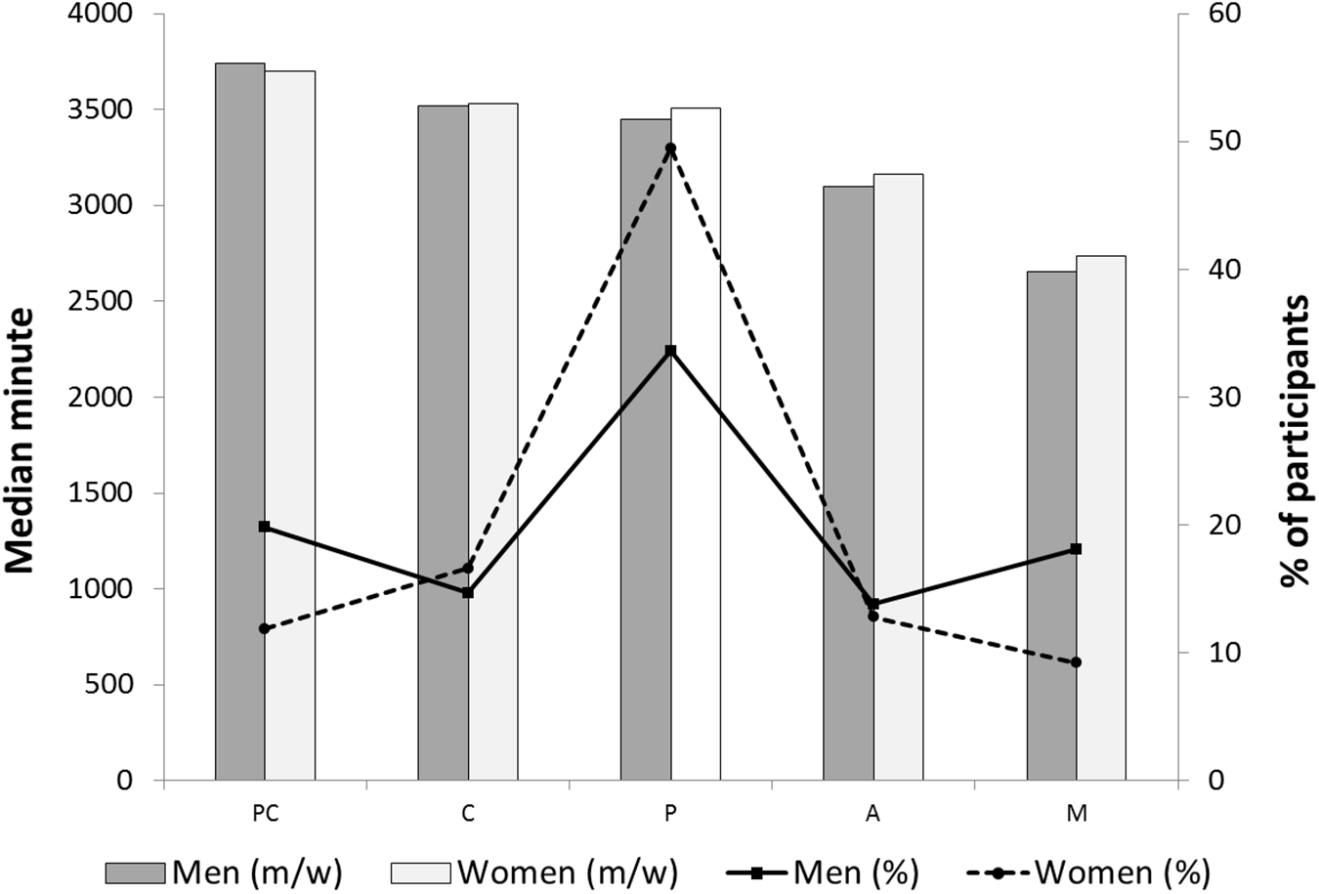
Stage distributions (%) and median minutes of sedentary time (m/w) for each stage by gender. PC = precontemplation; C = contemplation; P = preparation; A = action; M = maintenance.

The distributions of participants and unadjusted median minutes of sedentary behaviors across stages of motivational readiness to avoid sedentary behaviors by gender are shown in Fig 1. Among men, the greatest proportion of the participants was more likely to report being in the Preparation stage (33.6%) followed by the Precontemplation (19.8%) and Maintenance (18.1%) stages. A similar pattern was shown among women. The highest percentage of the female participants was in the Preparation stage (49.5%), but they were more likely to be in Contemplation (16.5%) and Action (12.8%) stages. Both sedentary times unadjusted and adjusted for wear time tended to be lower at higher stages for both men and women. Mann-Whitney U tests and independent-sample *t* tests revealed no significant differences between men and women in the unadjusted and adjusted times of sedentary behaviors for each stage as well as total sedentary time (*p* = 0.27 and 0.12, respectively) (Table 2). The median daily device wear times were 749.1 min/day (IQR 637.7–906.6) and 770.6 min/day (IQR 639.6–961.5) for men and women, respectively.

**Table 2:** Stage distributions and weekly median and mean sedentary minutes unadjusted and adjusted for wear time by gender.

Stage	Men			Women			Sig. (MWU/*t*)
	n (%)	Unadjusted Median (m/w [IQR])	Adjusted Mean (% (SD))	n (%)	Unadjusted Median (m/w [IQR])	Adjusted Mean (% (SD))	
PC	23 (19.8)	3741.7 [3267.5, 4148.6]	0.65 (0.07)	13 (11.9)	3702.7 [3267.5, 4148.6]	0.65 (0.07)	0.62/0.71
C	17 (14.7)	3521.2 [2649.8, 4578.9]	0.64 (0.09)	18 (16.6)	3529.3 [2874.0, 4165.2]	0.64 (0.05)	0.61/0.85
P	39 (33.6)	3448.8 [2651.9, 3996.6]	0.62 (0.09)	54 (49.5)	3505.7 [2824.2, 3886.5]	0.63 (0.08)	0.74/0.10
A	16 (13.8)	3100.4 [2709.8, 3589.5]	0.62 (0.05)	14 (12.8)	3164.7 [2587.0, 3698.7]	0.62 (0.07)	0.73/0.75
M	21 (18.1)	2655.5 [2333.1, 3031.1]	0.60 (0.06)	10 (9.2)	2739.2 [2220.2., 3352.5]	0.61 (0.07)	0.69/0.25
Total	116	3270.7 [2652.8, 3906.5]	0.63 (0.08)	109	3476.4 [2796.2, 4098.3]	0.64 (0.07)	0.27/0.12

*Note*. n = number of participants; IQR = Inter-quartile range; SD = standard deviation; MWU = Mann-Whitney U test; t = independent-sample *t* test; PC = precontemplation; C = contemplation; P = preparation; A = action; M = maintenance.

Mean scores, standard deviations, results of MANOVAs with *p*-values, and Bonferroni’s post hoc contrasts of the TTM core constructs for avoiding sedentary behaviors across the stages are shown in Table 3. Some processes (i.e., consciousness raising, environmental reevaluation, counter conditioning, self-liberation, and stimulus control) were used significantly more frequently by the participants in later stages than those in earlier stages in both genders. No significant differences were found across the stages for men or both men and women in other processes, such as dramatic relief, self-reevaluation, social liberation, contingency management, and helping relationships. In general, participants in the Precontemplation stage were less likely to use the processes to reduce sedentary behaviors than those in Preparation, Action, and/or Maintenance stages.

**Table 3:** Means of the TTM constructs with standard deviations across stages of motivational readiness for sedentary behaviors.

Construct	Gender	Stages of Motivational Readiness					F	Bonferroni’s post hoc	Ƞ_p_^2^
		PC	C	P	A	M			
Consciousness Raising	M	1.49 (0.51)	1.76 (0.74)	1.89 (0.73)	2.12 (0.82)	2.17 (0.83)	3.69**	PC < A/M	0.12
	W	1.48 (0.47)	1.83 (0.69)	2.06 (0.69)	2.06 (0.97)	2.14 (0.89)	2.34*	PC < M	0.08
Dramatic Relief	M	2.04 (0.85)	2.61 (0.94)	2.59 (0.87)	2.63 (1.02)	2.68 (0.86)	1.74		0.06
	W	1.98 (0.91)	2.63 (0.87)	2.75 (0.80)	2.65 (0.73)	2.86 (0.87)	3.18*	PC < P/M	0.11
Environmental Reevaluation	M	2.19 (0.82)	2.58 (0.83)	2.73 (0.77)	2.89 (0.98)	2.62 (0.87)	2.39*	PC < A	0.08
	W	1.90 (0.74)	2.62 (0.74)	2.83 (0.65)	2.55 (0.76)	2.92 (0.75)	5.18***	PC < P/M	0.17
Self-Reevaluation	M	2.88 (0.79)	3.24 (0.69)	3.22 (0.82)	3.01 (0.76)	3.03 (0.60)	1.73		0.06
	W	2.54 (1.30)	3.30 (0.86)	3.48 (0.76)	3.25 (0.74)	3.75 (0.87)	3.85**	PC < P/M	0.13
Social Liberation	M	2.13 (0.68)	2.59 (0.77)	2.60 (0.90)	2.64 (0.68)	2.72 (0.62)	2.21		0.07
	W	2.03 (0.66)	2.55 (0.85)	2.62 (0.75)	2.68 (0.86)	2.60 (0.55)	2.14		0.08
Contingency Management	M	2.26 (0.85)	2.54 (1.01)	2.74 (0.82)	2.72 (0.93)	2.92 (0.91)	1.78		0.06
	W	2.22 (0.85)	2.70 (0.93)	2.86 (0.82)	2.78 (0.91)	3.02 (1.01)	1.79		0.06
Counter Conditioning	M	2.52 (0.70)	2.96 (0.62)	3.13 (0.86)	3.39 (0.70)	3.57 (0.75)	7.15***	PC < A/M, C < M	0.20
	W	2.46 (0.73)	3.02 (0.84)	2.93 (0.78)	3.27 (0.82)	3.47 (0.96)	2.85*	PC < M	0.10
Helping Relationships	M	1.75 (0.64)	2.07 (0.56)	2.20 (0.67)	2.09 (0.84)	2.08 (0.71)	1.64		0.06
	W	1.75 (0.87)	1.79 (0.66)	2.08 (0.78)	2.25 (1.02)	2.26 (0.89)	1.43		0.05
Self-Liberation	M	2.48 (0.92)	3.30 (0.85)	3.15 (0.86)	3.28 (0.96)	3.46 (0.78)	3.91***	PC < C/M	0.12
	W	2.42 (0.99)	2.94 (0.64)	3.41 (0.69)	3.18 (1.01)	3.30 (0.89)	4.62***	PC < P	0.15
Stimulus Control	M	1.35 (0.37)	1.92 (0.55)	1.95 (0.88)	2.01 (0.61)	2.06 (0.63)	5.24***	PC < C/P/A/M	0.16
	W	1.35 (0.43)	1.92 (0.78)	1.99 (0.58)	2.07 (0.69)	2.12 (0.99)	2.83*	PC <M	0.10
Self-Efficacy	M	2.49 (0.49)	2.65 (0.77)	2.94 (0.79)	3.01 (0.65)	3.10 (0.70)	3.10*	PC < M	0.10
	W	2.19 (0.49)	2.89 (0.77)	2.98 (0.79)	2.83 (0.65)	3.02 (0.70)	3.17*	PC < M	0.11
Decisional Balance	M	0.13 (0.96)	0.69 (1.16)	1.02 (0.85)	1.39 (1.09)	1.44 (1.14)	5.83***	PC < P/A/M	0.17
	W	0.11 (0.87)	0.35 (0.67)	1.22 (1.02)	1.27 (0.79)	1.48 (1.28)	4.61***	PC < A/M	0.15

*Note*. PC = precontemplation; C = contemplation; P = preparation; A = action; M = maintenance.

* = *p* < 0.05

** = *p* < 0.01

*** = *p* < 0.001.

Self-efficacy to avoid sedentary behaviors was significantly different across the stages in both male and female participants (F[4,111] = 3.10, *p* = 0.019 and F[4, 104] = 3.17, *p* = 0.017, respectively). Post hoc analyses revealed that participants who reported being in the Maintenance stage had significantly higher self-efficacy to avoid sedentary behaviors than those in the Precontemplation stage for both men and women. In addition, the scores of decisional balance were significantly different between the stages in both men and women (F[4,111] = 5.83, *p* = 0.000 and F[4, 104] = 4.61, *p* = 0.002, respectively). Both male and female students in later stages perceived more pros and fewer cons for avoiding sitting times than did participants in the Precontemplation stage. Self-efficacy and decisional balance have indicated the above medium and large effects, respectively based on Cohen’s classification [28].

### Relationship between physical activity and sedentary behavior

The results of Chi-square tests and Spearman correlation for identifying the association between physical activity and sedentary behaviors are shown in Table 4. No significant associations were observed between the five stages of motivational readiness for avoiding sedentary behaviors and the two groups of meeting/non-meeting physical activity guidelines (i.e., based on accelerometer-derived physical activity time with cut-point of 150 min/week) in both men (*p* = 0.67) and women (*p* = 0.52). Consistent with accelerometer classification, the five stages were not significantly associated with the two groups of meeting/non-meeting the guidelines classified by self-reported questionnaire-derived leisure physical activity time with a cut-point of 7.5 MET·hour per week (i.e., *p* = 0.17 for men and *p* = 0.16 for women). In addition, Spearman correlations between total minutes of physical activity and sedentary behaviors measured by both an accelerometer and questionnaires showed no significant correlations between the two behaviors for both male and female students.

**Table 4:** Relationships between physical activity and sedentary behaviors.

Measurement	Gender	Chi-square Tests (Stages of SB x meeting/non-meeting PA guidelines)			Spearman Correlations (minutes of PA and sedentary behaviors)	
		χ^2^	df	Sig.	ρ	Sig.
Accelerometer	M	2.33	4	0.67	-0.07	0.44
	W	3.21	4	0.52	0.11	0.26
Questionnaire	M	6.46	4	0.17	-0.13	0.16
	W	6.53	4	0.16	-0.004	0.97

*Note*. SB = sedentary behaviors; PA = physical activity; M = men; W = women.

Comparisons of the TTM constructs by gender stratified by those meeting and not meeting physical activity guidelines are shown in Table 5. Of the 225 participants, 47.1% (n = 106) participated in physical activity sufficient to meet or exceed physical activity guidelines (accelerometer-derived estimates). No scores of the TTM constructs for avoiding sedentary behaviors were significantly different between the two groups except for consciousness raising (*p* = 0.01 for only women), dramatic relief (*p* = 0.04 and 0.02 for men and women, respectively), and social liberation (*p* = 0.03 for only women). In addition, more participants (n = 155, 68.9%) were meeting the guidelines when using questionnaire-derived total MET·hours per week of leisure physical activity. No significant differences were also found in the TTM constructs between the two groups except for counter conditioning for men (*p* = 0.03).

**Table 5:** Means and standard deviations for the TTM constructs for sedentary behaviors between the two groups of meeting and non-meeting physical activity guidelines.

Variable	Gender	Accelerometer					Questionnaire				
		Meeting n = 134 (59.6%)		Non-meeting n = 91 (40.4%)		Sig.	Meeting n = 155 (68.9%)		Non-meeting n = 70 (31.1%)		Sig.
		Mean	SD	Mean	SD		Mean	SD	Mean	SD	
Consciousness Raising	M	1.79	0.71	2.03	0.83	0.09	1.98	0.83	1.76	0.63	0.17
	W	2.16	0.70	1.80	0.75	0.01*	2.07	0.75	1.78	0.72	0.06
Dramatic Relief	M	2.36	0.91	2.71	0.93	0.04*	2.55	0.91	2.51	1.00	0.81
	W	2.82	0.79	2.44	0.88	0.02*	2.70	0.85	2.47	0.87	0.18
Environmental Reevaluation	M	2.55	0.87	2.62	0.85	0.67	2.58	0.80	2.61	1.01	0.84
	W	2.75	0.74	2.59	0.77	0.27	2.64	0.76	2.72	0.75	0.63
Self-Reevaluation	M	3.08	0.73	3.18	0.80	0.47	3.12	0.71	3.16	0.89	0.82
	W	3.37	0.90	3.27	0.92	0.55	3.32	0.96	3.32	0.81	0.96
Social Liberation	M	2.42	0.77	2.64	0.72	0.12	2.52	0.76	2.57	0.76	0.75
	W	2.71	0.79	2.38	0.78	0.03*	2.58	0.81	2.44	0.79	0.39
Contingency Management	M	2.48	0.76	2.81	0.99	0.06	2.62	0.84	2.71	1.04	0.63
	W	2.88	0.96	2.64	1.06	0.23	2.85	1.00	2.57	1.05	0.17
Counter Conditioning	M	3.07	0.74	3.07	0.82	0.95	3.17	0.77	2.82	0.75	0.03*
	W	3.10	0.85	2.95	0.82	0.33	3.08	0.86	2.90	0.79	0.28
Helping Relationships	M	1.93	0.70	2.18	0.67	0.06	2.07	0.68	2.03	0.72	0.79
	W	1.99	0.82	2.13	0.83	0.39	2.06	0.80	2.07	0.89	0.98
Self-Liberation	M	2.92	0.95	3.19	0.87	0.11	3.10	0.84	2.99	1.09	0.56
	W	3.30	0.89	3.07	0.78	0.16	3.20	0.89	3.15	0.75	0.80
Stimulus Control	M	1.73	0.60	1.96	0.66	0.06	1.87	0.63	1.79	0.67	0.56
	W	2.00	0.75	1.82	0.62	0.16	1.93	0.71	1.86	0.64	0.65
Self-Efficacy	M	2.75	0.75	2.89	0.76	0.33	2.85	0.68	2.76	0.93	0.58
	W	2.90	0.78	2.81	0.75	0.54	2.90	0.74	2.76	0.81	0.36
Decisional Balance	M	0.85	1.04	1.08	1.14	0.26	1.01	1.05	0.88	1.21	0.57
	W	1.16	1.08	1.06	1.09	0.62	1.20	1.05	0.92	1.12	0.20

*Note*. n = number of participants; SD = standard deviation; M = men; W = women.

* = *p* < 0.05.

## Discussion

The present study examined college students’ sedentary behaviors using a modified Transtheoretical Model for avoiding sedentary behaviors and investigated how the TTM outcomes are associated with current physical activity levels. The results indicate that a majority of the participants among both men and women perceive that they are currently sedentary most of the day, but intend to reduce their sitting times soon or occasionally try to interrupt prolonged sitting time. In addition, some processes are used more frequently in later stages to avoid sedentary behaviors, and both male and female students in later stages tend to be more confident to avoid sedentary behaviors and to perceive more pros and fewer cons for reducing sedentary behaviors than individuals in earlier stages. Lastly, individuals’ current physical activity levels are not significantly associated with their TTM outcomes regarding sedentary behaviors.

An important finding from the current study is that the stage distributions for avoiding sedentary behaviors were different in male and female students. For example, generally, more female students were in sedentary stages (e.g., Precontemplation, Contemplation, or Preparation) than male students. These findings concur with previous studies in which females were more sedentary than males in early adulthood [5]. However, among the stages, more men tended to be in the Precontemplation stage than women, whereas more women tended to be in the Preparation stage than men. This result suggested that although women tend to be more sedentary than men, women may have a higher likelihood of subsequent changes in sedentary behaviors than men (e.g., stage progression to the Action or Maintenance stages) while men may have more potential to progress within the early stages (e.g., strengthened intentions or improved motivation without actual change in sedentary behaviors) than women. In addition, a higher percentage of men was found to be in the Maintenance stage compared to women. These results revealed that relative to women, men are not only less likely to intend to avoid sitting time, but men are also more likely to perform frequent movements to avoid or break prolonged sitting time. These findings are comparable to those in a study of 493 college students that the male students reported more sedentary times spent in watching television and/or video and using the computer compared to female students (*p* < .05). Concurrently, the male students self-reported a higher amount of exercise compared to female students on most of the exercise indicators (*p* < .05) [29].

Men and women also differed in means of the TTM constructs for sedentary behaviors across the stages. In general, both men and women in later stages had higher means of the TTM constructs than those in earlier stages. For processes of change, this finding is consistent with previous studies indicating people need to use more processes of change to progress to later stages [30]. However, the most frequently used processes for changing sedentary behaviors were different by gender. For instance, dramatic relief, environmental reevaluation, self-reevaluation, and self-liberation were examples of the specific processes that were most frequently used to change sedentary behaviors by women whereas men tended to mostly use consciousness raising, counter conditioning, and stimulus control. These findings may provide important information on choosing relevant strategies for changing sedentary behaviors among male and female college students in future research. Indeed, other findings regarding self-efficacy and decisional balance in the present study support the idea that self-efficacy and decisional balance for various behaviors increases as progressing across the stages [31, 32]. Our results suggested that both constructs of self-efficacy and decisional balance for sedentary behaviors can be employed as a useful strategy for future intervention research. Therefore, the findings regarding the TTM for avoiding sedentary behaviors highlighted the gender differences in determinants of sedentary behaviors and emphasized the importance of tailored-interventions for individuals within each stage to reduce sedentary behaviors.

To the authors’ knowledge, this is the first study investigating the associations between the theory-based determinants of sedentary behaviors (i.e., TTM outcomes for sedentary behaviors) and physical activity levels (i.e., meeting or non-meeting physical activity guidelines). Our findings indicated that no significant associations were found between TTM outcomes for sedentary behaviors and individual’s physical activity levels in both genders. A consistent finding was also found in the current study that there were no correlations between the minutes of physical activity and sedentary behaviors in both men and women. These findings are in agreement with many previous investigations reporting little relationship between the amount of time spent in sedentary behaviors and the time spent in physical activity [29, 33]. However, it should be highlighted that the current study used objective measurements of physical activity and sitting time to determine the relationship, which might address the limitations of the previous studies relying on self-reports of television viewing and physical activity [29, 33]. In addition, the present study investigated the TTM-based determinants of sedentary behaviors in relation to physical activity levels which is distinct from many previous studies indicating an association between the determinants of physical activity and screen time [34]. Therefore, the results of this study support the argument that physical activity and sedentary behaviors are two independent behaviors, not simple opposites [33], with a couple different approaches.

The strengths of the current study include the first approach of examining sedentary behaviors using the TTM and of investigating the association between determinants of sedentary behavior and current physical activity levels. Our results may provide useful estimates of theory-based constructs and unique information about the association between the two behaviors. An additional strength of this study was the use of multiple tests and measurements in order to verify the association between physical activity and sedentary behaviors. For example, both objective (e.g., accelerometer) and self-reported (e.g., questionnaire) measurements were used to estimate the times spent in physical activity and sedentary behaviors. Also, multiple tests such as a Chi-square test, Spearman correlation, and MANOVAs were conducted to identify the associations. Perhaps, these processes strengthen the results of the present study.

Several limitations of this study should also be considered. Study participants were composed of a convenience sample of college students enrolled in health education or physical education courses. This may limit the generalizability of the findings to diverse populations. Further, the present study includes the nature of the cross-sectional study design which is unable to confirm stage changes over time and precludes causal inferences for the association between physical activity and sedentary behaviors. Finally, self-reported TTM questionnaires may misclassify the participants into incorrect stages or cause recall bias. However, the TTM questionnaires have reported high scores of validity and reliability among the same population [19], and the objectively measured sedentary times support the stage classification.

The current study describes college students’ sedentary behaviors based on the TTM and the association between the TTM outcomes and physical activity levels. Overall, a majority of the participants perceived their sedentary behaviors and intended to change their sedentary behaviors. When changing sedentary behaviors, men and women tended to use different strategies. Further, their sedentary behaviors were not associated with current physical activity levels. The results from the current study suggest that researchers and practitioners should consider physical activity promotion and sedentary behavior reduction as two independent intervention goals instead of conflicting behaviors.

## Supporting information

S1 Questionnaire (PDF)

S1 Table Summary estimates of sedentary time and physical activity unadjusted and adjusted for total device wear times. (DOCX)
