# Supplementary material for: Application of the transtheoretical model to sedentary behaviors and its association with physical activity status
Source: PLoS One. 2017 Apr 27;12(4):e0176330. doi: 10.1371/journal.pone.0176330 (PMC5407750; doi:10.1371/journal.pone.0176330)
Supplement: S1 Questionnaire — (PDF) [file pone.0176330.s001.pdf]

UNIVERSITY STUDENT PHYSICAL ACTIVITY & SEDENTARY BEHAVIOR QUESTIONNAIRES

Thank you for participating in our study. Your answers are very important, so please be as accurate as you can while answering. All numbers should be to the nearest whole number.

DEMOGRAPHIC INFORMATION

Name: ______________________

EID: ________________________

FOR STAFF USE ONLY

Group #: ____________________

Participant ID: ________________

1. Age (yr) ______________
2. Sex ______________
3. Height _______feet _______inches
4. Weight ____________Lbs
5. Ethnicity ______________
6. The college year ______________
7. Major ____________________
8. Number of physical education course you have taken in college ______________
9. Number of physical education course you are currently taking in 2013 Fall ______________

   If taking more than one, please list the course names ____
10. Exercise preferences ____
11. Time spent in additional weight training outside of class per week ________________minutes
12. Have you seen, heard, or read about the existence of government physical activity guidelines?

    Yes ☐ No ☐ Not sure ☐
13. What is the minimum amount of physical activity the government recommends for adult to get overall health benefits? (e.g., duration (min), and frequent (days/week))

TRANSTHEORETICAL MODEL QUESTIONNAIRE FOR SEDENTARY BEHAVIOR

1. Stages of Motivational Readiness to Avoid Sitting Time

Instructions: Researchers are investigating the role of sitting time on health outcomes. ***Sitting time*** is defined as ***any time that outside of sleep during which you are sitting or reclining and experience less than 1.5 METs of physical activity***. Examples include: **sitting, studying, watching TV, playing video games, using computer, etc.** Some people try to limit or reduce their sitting time by using a standing desk, taking walk or stretch breaks, etc. When answering the questions below, consider prolonged periods of sitting behavior that you normally experience. Please be sure to read the questions carefully and answer the questions by checking one of the following five cases that ***best describes your intention.***

Question 1) Do you achieve sufficient levels of physical activity on most days? Yes ☐ No ☐

Question 2) Do you think you are currently sitting most of the day?

1. Yes, and I do not intend to avoid my sitting time ☐
2. Yes, but I intend to avoid my sitting time *within the next 6 months.* ☐
3. Yes, but I intend to avoid my sitting time *within the next 30 days* or sometimes do some movements such as interruption (break) of prolonged sitting to reduce sitting time. ☐
4. No, I am not sitting. I began doing frequent movements to interrupt or avoid prolonged sitting time *within the last 6 months.* ☐
5. No, I am not sitting. I began doing frequent and regular movements to avoid or break prolonged sitting time *more than 6 months ago* ☐

2. Processes of Changes for Avoiding Sitting Time

Instructions: Researchers are investigating the role of sitting time on health outcomes. ***Sitting time*** is defined as ***any time that outside of sleep during which you are sitting or reclining and experience less than 1.5 METs of physical activity***. Examples include: **sitting, studying, watching TV, playing video games, using computer, etc.** Some people try to limit or reduce their sitting time by using a standing desk, taking walk or stretch breaks, etc. When answering the questions below, consider prolonged periods of sitting behavior that you normally experience.

The following experiences can affect the sitting habits of some people. Think of any similar experiences you may currently have or have had during the **past month**. Then ***rate how frequently the behavior occurs***. Please ***circle the number that best describes your answer for each experience***.

Scale

1 = Never, 2 = Seldom, 3 = Occasionally, 4 = Often, 5 = Repeatedly

**1.** I feel more competent myself when I decide to avoid sitting time	1 2 3 4 5
**2.** I worry that prolonged periods of sitting behaviors can be harmful to my body	1 2 3 4 5
**3.** I think about information from articles and advertisements on how to decrease sitting time	1 2 3 4 5
**4.** I tell myself that if I try hard enough, I can avoid sitting behaviors	1 2 3 4 5
**5.** When I have free time, I look for some alternate activities to do instead of sitting behaviors, such as playing game or watching TV/movies	1 2 3 4 5
**6.** I see advertisements on television about how society is trying to help people to reduce sitting time	1 2 3 4 5
**7.** I wonder how my sitting lifestyle affects those people who are close to me	1 2 3 4 5
**8.** I have someone who can help me to avoid sitting lifestyle	1 2 3 4 5
**9.** I stay away from places generally associated with sitting behaviors	1 2 3 4 5
**10.** I can expect to be praised by others if I try to reduce sitting time	1 2 3 4 5
**11.** I make commitments to avoid sitting time	1 2 3 4 5
**12.** I put things around my place or work that remind me to reduce sitting time	1 2 3 4 5
**13.** I notice that school environments such as sport facilities or physical education programs are encouraging students to reduce sitting time	1 2 3 4 5

1 = Never, 2 = Seldom, 3 = Occasionally, 4 = Often, 5 = Repeatedly	
**14.** Some of my close friends or family members might be more sitting if I were more sitting	1 2 3 4 5
**15.** I seek newspaper stories or articles about reducing sitting time in an attempt to learn	1 2 3 4 5
**16.** Remembering studies about illnesses caused by sitting lifestyle makes me worry	1 2 3 4 5
**17.** When I am avoiding sitting time, I tell myself that I am being good to myself by taking care of my body	1 2 3 4 5
**18.** I have someone who provides feedback about my sitting lifestyle	1 2 3 4 5
**19.** I am considering the idea that reducing my sitting time would make me a healthier, happier person	1 2 3 4 5
**20.** When I am tempted to be sitting, I think about something else to avoid sitting behaviors	1 2 3 4 5
**21.** Dramatic portrayals of the consequences of sitting lifestyle affect me emotionally	1 2 3 4 5
**22.** I am aware of more and more people encouraging me to decrease sitting time these days	1 2 3 4 5
**23.** I have someone who points out my rationalizations for being sedentary	1 2 3 4 5
1 = Never, 2 = Seldom, 3 = Occasionally, 4 = Often, 5 = Repeatedly	
**24.** I remove things that contribute to my sitting behaviors	1 2 3 4 5
**25.** I recall information people have personally given me on the benefits of avoiding sitting time	1 2 3 4 5
**26.** I become disappointed with myself when I think about my sitting lifestyle	1 2 3 4 5
**27.** I do something else instead of being sitting when I need to relax or deal with tension	1 2 3 4 5
**28.** I think that I might be able to influence others to be healthier if I would change my sitting lifestyle	1 2 3 4 5
**29.** I do something nice for myself for making efforts to reduce sitting time	1 2 3 4 5
**30.** I am considering that I am the only person responsible for my health, and only I can decide whether or not I will avoid sitting behaviors	1 2 3 4 5
**31.** Warnings about the health hazards of sitting lifestyle cause me to feel badly	1 2 3 4 5
**32.** I notice society changing in ways that help to reduce sitting time	1 2 3 4 5
**33.** I have a friend who encourages me to avoid sitting time when I am	1 2 3 4 5
**34.** I feel that I would be a better role model for others if I reduced my sitting behaviors	1 2 3 4 5

1 = Never, 2 = Seldom, 3 = Occasionally, 4 = Often, 5 = Repeatedly	
**35.** I tell myself that I need to reduce sitting time	1 2 3 4 5
**36.** I look for information related to problems associated with sitting behaviors	1 2 3 4 5
**37.** I remove things from my home or work that remind me to be sitting	1 2 3 4 5
**38.** I think about the type of person I would be if I were not sedentary	1 2 3 4 5
**39.** Instead of remaining sedentary, I engage in some physical activities	1 2 3 4 5
**40.** I praise or reward myself when I try to avoid sitting behaviors	1 2 3 4 5

3. Self-Efficacy

Instructions: Researchers are investigating the role of sitting time on health outcomes. ***Sitting time*** is defined as ***<u>any time that outside of sleep during which you are sitting or reclining and experience less than 1.5 METs of physical activity</u>***. Examples include: **sitting, studying, watching TV, playing video games, using computer, etc.** Some people try to limit or reduce their sitting time by using a standing desk, taking walk or stretch breaks, etc. When answering the questions below, consider prolonged periods of sitting behavior that you normally experience. Please be sure to read the questions carefully and answer the questions by checking one of the following five cases that ***best describes your intention.***

<u>Listed below are situations that lead some people to be sitting. We would like to know **how confident you are** that ***you can avoid or break (> 1min) prolonged sitting time*** in each of the following situations. Please ***circle the number that best describes your answer for each situation***.</u>

Scale
1 = Not at all confident
2 = Somewhat confident
3 = Moderately confident
4 = Very confident
5 = Extremely confident

1. When I am doing schoolwork (e.g., study or assignments)	1 2 3 4 5
2. When I am feeling tired	1 2 3 4 5
3. When I am playing video games, using computer or watching TV	1 2 3 4 5
4. When I am spending time with friends or family who want to be sitting	1 2 3 4 5
5. When I am on vacation or have free time	1 2 3 4 5
6. When I am feeling lazy	1 2 3 4 5

4. Decisional Balance

Instructions: Researchers are investigating the role of sitting time on health outcomes. ***Sitting time*** is defined as ***any time that outside of sleep during which you are sitting or reclining and experience less than 1.5 METs of physical activity***. Examples include: **sitting, studying, watching TV, playing video games, using computer, etc.** Some people try to limit or reduce their sitting time by using a standing desk, taking walk or stretch breaks, etc. When answering the questions below, consider prolonged periods of sitting behavior that you normally experience. Please be sure to read the questions carefully and answer the questions by checking one of the following five cases that ***best describes your intention.***

This section looks at positive and negative aspects of sitting behaviors. Read the following items carefully and indicate ***how important each statement is with respect to your decision of whether to avoid sitting time or not***. In each case, think about how you feel **right now**, not how you have felt in the past or would like to feel. Please ***circle the number that best describes your answer for each case***.

Scale
1= Not at all important
2 = Slightly important
3 = Moderately important
4 = Very important
5 = Extremely important

**1.** Reducing sitting time would make me have a more positive outlook on life	1 2 3 4 5
**2.** Reducing sitting time would make me tired for the rest of the day	1 2 3 4 5
**3.** Avoiding sitting time would be beneficial for my health	1 2 3 4 5
**4.** I would feel lazy doing something to avoid sitting time	1 2 3 4 5
**5.** I would feel more diligent with avoiding sitting time	1 2 3 4 5
**6.** Breaking sitting time would disrupt my concentration when I am studying	1 2 3 4 5
**7.** I would feel proud of myself if people saw my efforts to avoid sitting time and consider me as a role model	1 2 3 4 5
**8.** Spending time in sitting is more comfortable for me	1 2 3 4 5
**9.** Avoiding sitting time would make me active person	1 2 3 4 5
**10.** I would feel I wasted my energy by reducing sitting time	1 2 3 4 5
**11.** Avoiding sitting time would help to refresh my mind and body	1 2 3 4 5
**12.** I would feel uncomfortable avoiding sitting time in public place. For example standing in the library reading or standing while watching TV with friends	1 2 3 4 5

MODIFIABLE ACTIVITY QUESTIONNAIRE

We would like to learn more about the type(s) of physical activity you did in the past seven (7) days.

Let's get started!

Instructions:

First, enter today's date and day of the week in the first (**DAY 0**) column. Next, repeat this process by entering the date and day, counting backwards from **1 DAY AGO** (*yesterday*) to **7 DAYS AGO** (See Example).

	1. Enter the date and day of the week for **TODAY (DAY 0)** back to **7 DAYS AGO.**							
	**TODAY DAY 0**	**1 DAY AGO**	**2 DAYS AGO**	**3 DAYS AGO**	**4 DAYS AGO**	**5 DAYS AGO**	**6 DAYS AGO**	**7 DAYS AGO**
**EXAMPLE →**	1-11-13	1-10-13	1-9-13	1-8-13	1-7-13	1-6-13	1-5-13	1-4-13
	Tues	Mon	Sun	Sat	Fri	Thurs	Wed	Tues
**DATE →**								
**DAY →**								

When filling out this survey, we'd like you to think about the physical activities you did for *10 minutes or longer* from **1 DAY AGO** (*yesterday*) to **7 DAYS AGO.**

The chart below lists activities that you may do during your **spare time or for exercise**. Start with "Aerobic Dance or Step Aerobics" and work down the list of activities.

If you **did not do the activity**, check the **"NO"** box and move down the list to the next activity (See Example Activity 1). If you **did the activity,** check the **"YES"** box and enter the number of minutes that you did the activity each day. If you did not do the activity on a particular day, please enter a "0" for that day (See Example Activity 2).

2. In the past seven (7) days did you do any of the following activities **during your spare time**?	**NO** ↓	**YES** ↳	3. If **"YES"**, enter the total number of minutes (mins) you did the activity on each day. *If you didn't do the activity on a day, enter a "0".*						
			**1 DAY AGO mins**	**2 DAYS AGO mins**	**3 DAYS AGO mins**	**4 DAYS AGO mins**	**5 DAYS AGO mins**	**6 DAYS AGO mins**	**7 DAYS AGO mins**
**Example Activity 1**	☒	☐							
**Example Activity 2**	☐	☒	30	0	30	0	0	30	0
Aerobic Dance or Step Aerobics	☐	☐							
Badminton	☐	☐							

2. In the past seven (7) days did you do any of the following activities **during your spare time**?	NO	YES	3. If **"YES"**, enter the total number of minutes (mins**)** you did the activity on each day. *If you didn't do the activity on a day, enter a "0".*						
			1 DAY AGO	2 DAYS AGO	3 DAYS AGO	4 DAYS AGO	5 DAYS AGO	6 DAYS AGO	7 DAYS AGO
			mins	mins	mins	mins	mins	mins	mins
Basketball	☐	☐							
Bicycling for Exercise	☐	☐							
Bowling	☐	☐							
Calisthenics or Toning Exercises	☐	☐							
Canoeing, Rowing, or Kayaking	☐	☐							
Dancing (e.g., line, square, ballroom)	☐	☐							
Elliptical Trainer	☐	☐							
Fencing	☐	☐							
Fishing	☐	☐							
Football or Soccer	☐	☐							
Gardening or Yard work	☐	☐							
Golf	☐	☐							
Hiking	☐	☐							
Horseback Riding	☐	☐							
Hunting	☐	☐							
Jogging (indoor or outdoor)	☐	☐							
Jumping Rope	☐	☐							
Martial Arts (e.g., karate, judo)	☐	☐							
Pilates	☐	☐							
Racquetball, Handball, or Squash	☐	☐							
Rock Climbing	☐	☐							
Scuba Diving	☐	☐							
Skating (e.g., roller, ice, or roller-blading)	☐	☐							
Snow shoeing	☐	☐							
Snow skiing (downhill)	☐	☐							
Snow skiing (cross country or Nordic track)	☐	☐							

2. In the past seven (7) days did you do any of the following activities **during your spare time**?	NO	YES	3. If **"YES"**, enter the total number of minutes (mins**)** you did the activity on each day. *If you didn't do the activity on a day, enter a "0".*						
			**1 DAY AGO** mins	**2 DAYS AGO** mins	**3 DAYS AGO** mins	**4 DAYS AGO** mins	**5 DAYS AGO** mins	**6 DAYS AGO** mins	**7 DAYS AGO** mins
Softball or Baseball	☐	☐							
Stairmaster	☐	☐							
Strength or Weight Training	☐	☐							
Swimming (laps or snorkeling)	☐	☐							
Tai Chi	☐	☐							
Tennis	☐	☐							
Walking for Exercise	☐	☐							
Water Aerobics	☐	☐							
Yoga	☐	☐							
Other ____________	☐	☐							

Continued on next page

The second chart below lists activities that you may do to **travel from one place to another**. We'd like you to think of each trip as a **one-way trip**. For each trip, enter the number of minutes that you were physically active during travel. If you did not do the activity on a particular day, please enter a "0" for that day (See Example Activity 1).

4. In the past seven (7) days did you do any of the following activities **for transportation**?	NO	YES		5. If **"YES"**, enter the total number of minutes (mins) you did the activity on each day. *If you didn't do the activity on a day, enter a "0".*						
				**1 DAY AGO**	**2 DAYS AGO**	**3 DAYS AGO**	**4 DAYS AGO**	**5 DAYS AGO**	**6 DAYS AGO**	**7 DAYS AGO**
**Example Activity 1**	☐	☒		**mins**	**mins**	**mins**	**mins**	**mins**	**mins**	**mins**
			One-Way Trip 1	15	10	0	0	15	0	0
			One-Way Trip 2	10	20	0	0	5	0	0
			One-Way Trip 3	0	0	0	0	20	0	0
			One-Way Trip 4	0	0	0	0	0	0	0
Walking for Transport	☐	☐		**mins**	**mins**	**mins**	**mins**	**mins**	**mins**	**mins**
			One-Way Trip 1							
			One-Way Trip 2							
			One-Way Trip 3							
			One-Way Trip 4							
Bicycling for Transport	☐	☐		**mins**	**mins**	**mins**	**mins**	**mins**	**mins**	**mins**
			One-Way Trip 1							
			One-Way Trip 2							
			One-Way Trip 3							
			One-Way Trip 4							
Other Activity (e.g., skateboarding) for Transport	☐	☐		**mins**	**mins**	**mins**	**mins**	**mins**	**mins**	**mins**
			One-Way Trip 1							
			One-Way Trip 2							
			One-Way Trip 3							
			One-Way Trip 4							

Continued on next page

	NO	YES	
6. Was this week reflective of your usual physical activity levels?	☐	☐	If **NO**, why not? ________________
7. In the past seven (7) days, have you spent more than one day in a bed or chair as a result of an injury, illness or surgery?	☐	☐	If **YES**, how many days? ________**DAYS**
8. In the past seven (7) days, did you have difficulty doing any of the following activities?			
a. Getting in or out of a bed or chair?	☐	☐	
b. Walking across a small room without resting?	☐	☐	
c. Walking for 10 minutes without resting?	☐	☐	
9. In the past seven (7) days, excluding time at work, did you:			
a. Watch television?	☐	☐	If **YES**, how many hours typically per day? ________**HOURS PER DAY**
b. Play video games?	☐	☐	If **YES**, how many hours typically per day? ________**HOURS PER DAY**
c. Use the computer or internet?	☐	☐	If **YES**, how many hours typically per day? ________**HOURS PER DAY**

10. Please check the box that corresponds to the current month of the year.

☐	☐	☐	☐	☐	☐	☐	☐	☐	☐	☐	☐
**JAN**	**FEB**	**MAR**	**APR**	**MAY**	**JUN**	**JUL**	**AUG**	**SEPT**	**OCT**	**NOV**	**DEC**

MULTI-CONTEXT SITTING TIME QUESTIONNAIRE

1. Think about last 7 days. In the table below, please indicate the total number of hours and minutes per day you spend doing each of the activities listed. There is space to answer for a typical weekdays and weekends. Please try to remember as specifically as possible for each type of day. Please do not record time twice in different categories: for example, if you were reading while watching TV, only report that time under one category.

	Weekdays	Weekends
Sleeping	_____ hr _____min	_____ hr _____min
Sitting while using a computer or video game	_____ hr _____min	_____ hr _____min
Sitting while watching TV or movies	_____ hr _____min	_____ hr _____min
Sitting during transportation (not including bicycles)	_____ hr _____min	_____ hr _____min
Sitting while working, reading, or studying	_____ hr _____min	_____ hr _____min
Sitting while talking, texting, or other socializing	_____ hr _____min	_____ hr _____min

2. Do the answers to #1 reflect your normal activity levels? ☐Yes ☐No

3. At any time during the past 2 weeks, was your ability to move reduced due to injury or illness?

☐Yes ☐No

Thank you for taking the time to complete this survey!
