# Supplementary material for: Application of the transtheoretical model to sedentary behaviors and its association with physical activity status
Source: PLoS One. 2017 Apr 27;12(4):e0176330. doi: 10.1371/journal.pone.0176330 (PMC5407750; doi:10.1371/journal.pone.0176330)
Supplement: S1 Table — (DOCX) [file pone.0176330.s002.docx]

S2 Table. Summary estimates of sedentary time and physical activity unadjusted and adjusted for total device wear times

| Variable | Men (n=116) | Women (n=109) | Total (n=225) |
| --- | --- | --- | --- |
| Unadjusted | | | |
| Sedentary Time (min/d) | 467.2 [379.0, 558.1] | 496.6 [399.5, 585.5] | 486.4 [391.4, 570.9] |
| Bouted MVPA (min/d) | 24.9 [14.4, 39.6] | 24.2 [13.0, 35.9] | 24.4 [13.9, 38.2] |
| Accumulated Moderate-Intensity PA (min/d) | 64.1 [53.9, 76.6] | 57.5 [49.1, 69.4] | 61.2 [51.7, 71.6] |
| Accumulated Vigorous-Intensity PA (min/d) | 6.2 [4.1, 11.2] | 5.5 [3.0, 7.8] | 5.8 [3.5, 9.0] |
| Adjusted | | | |
| Sedentary Time (%) | 63.1 [57.2, 68.4] | 64.6 [60.3, 69.4] | 64.1 [58.9, 68.9] |
| Bouted MVPA (%) | 3.4 [1.9, 5.6] | 3.0 [1.6, 4.8] | 3.2 [1.6, 5.4] |
| Accumulated Moderate-Intensity PA (%) | 8.5 [6.7, 10.8] | 7.7 [6.2, 9.6] | 8.2 [6.4, 10.3] |
| Accumulated Vigorous-Intensity PA (%) | 0.8 [0.5, 1.5] | 0.7 [0.4, 1.1] | 0.7 [0.5, 1.4] |

*Note.* n = number of participants, MVPA = moderate to vigorous intensity physical activity, PA = physical activity

All values are medians with interquartile ranges
